# FDC-SP as a diagnostic and prognostic biomarker and modulates immune infiltrates in renal cell carcinoma

**DOI:** 10.1186/s12859-023-05215-1

**Published:** 2023-03-10

**Authors:** Fan Chang, Jiang-Hui Zhang, Wen-Song Wu, Shuai Tang, Zheng lv, Fang-Min Chen

**Affiliations:** 1 Department of Urology, The Third Central Clinical College of Tianjin Medical University, Tianjin, 300170 China; 2 Department of Urology, The Third Central Hospital of Tianjin, 83 Jintang Road, Hedong District, Tianjin, 300170 China; 3 Department of Urology, Nankai University Affinity the Third Central Hospital, Tianjin, 300170 China

**Keywords:** FDC-SP, RCC, Biomarker, Diagnosis, Prognosis, Immune infiltration

## Abstract

**Background:**

Renal cell carcinoma (RCC), one of the top 10 causes of cancer death, is responsible for more than 90% of all cases of primary renal cancer worldwide. Follicular dendritic cell-secreted protein (FDC-SP) specifically binds to activated B cells and regulates the generation of antibodies. It is also thought to promote cancer cell invasion and migration, which could help with tumor metastases. This study aimed to assess the efficacy of FDC-SP in the diagnosis and prognosis of RCC and to investigate the relationship between immune infiltration in RCC and these outcomes.

**Results:**

RCC tissues had significantly higher levels of FDC-SP protein and mRNA than normal tissues. The high level of FDC-SP expression was linked to the T stage, histological grade, pathological stage, N stage, M stage, and OS event. Functional enrichment analysis identified the major pathways that were enriched as immune response regulation, complement, and coagulation. Immunological checkpoints and immune cell infiltration were observed to substantially correlate with the levels of FDC-SP expression. FDC-SP expression levels showed the ability to precisely distinguish high-grade or high-stage renal cancer (area under the curve (AUC) = 0.830, 0.722), and RCC patients with higher FDC-SP expression levels had worse prognoses. The AUC values for one-, two-, and five-year survival rates were all greater than 0.600. Moreover, the FDC-SP expression is an independent predictive biomarker of OS in RCC patients.

**Conclusion:**

FDC-SP may be a prospective therapeutic target in RCC as well as a possible diagnostic and prognostic biomarker associated with immune infiltration.

**Supplementary Information:**

The online version contains supplementary material available at 10.1186/s12859-023-05215-1.

## Background

Malignant tumors of the urinary system, including renal carcinoma, account for 2% of adult malignancies [1, 2]. Renal cell carcinoma (RCC) is the most common type of renal cancer, and clear cell carcinoma is the most common pathological variety, accounting for over 75% of all primary renal malignancies [3]. RCC is difficult to diagnose in its early stages since there aren't any obvious symptoms, and in one-third of patients, distant metastases are already present before the tumor is discovered. Additionally, metastatic renal tumors arise from 20 to 40% of locally progressed renal cancers [4]. The lack of effective early diagnostic biomarkers contributes to the high mortality rates among RCC patients. The hematological tests that are now utilized to identify RCC non-invasively have inadequacies as well. The standard of care for localized kidney carcinoma is still radical or partial resection. However, 20–30% of patients will relapse after surgery [5]. Patients with advanced or metastatic renal cancer are typically treated with targeted pharmaceutical therapy and immunotherapy because renal cancer is resistant to radiation and chemotherapy. Tyrosine kinase inhibitors (TKIs) and mammalian target of rapamycin (mTOR) inhibitors make up a large percentage of targeted medications. The prognosis of patients can be improved by targeted drugs, but 20% of patients will have congenital drug resistance, and most patients will develop secondary drug resistance after 6–11 months [6, 7]. The cornerstones of immunotherapy are immunosuppressants that target the cytotoxic T-lymphocyte antigen-4 (CTLA-4) and programmed cell death 1 (PD-1) receptors. They can increase tumor recurrence reduction, increase patient survival, and strengthen the body's anti-tumor defenses. However, immunotherapy is more expensive, has more complicated treatment options, and causes adverse reactions that affect all organ systems throughout the body [8]. The prognosis of renal cancer is unknown because of the cancer's variable biological behavior and clear heterogeneity. Currently, clinical markers such as the clinical stage of tumors, the pathological grade, size, and other factors are still the primary factors used to determine the prognosis of renal cancer. Numerous molecular indicators associated with the effectiveness and prognosis of renal cancer have been found as a result of the widespread use of targeted medication therapy and immunotherapy for the treatment of renal cancer, offering fresh perspectives on the disease's management and prognosis monitoring. This requires us to simultaneously look for more sensitive molecular markers to identify tumors early, assess the effectiveness and prognosis of renal cancer treatment, and give patients more precise, individualized care.

The human gene for follicular dendritic cell-secreted protein (FDC-SP) is located on chromosome 4 and is primarily expressed in mucosal tissues [9]. FDC-SP is a unique secreted peptide with a distinctive immune system expression pattern and the ability to preferentially connect to activated B cells, according to past investigations. It might have an impact on autoimmune conditions and modify the B-cell immune response [10]. We discovered that FDC-SP could alter the expression of genes related to osteogenesis in periodontal disease while increasing the expression of genes related to osteoclastogenesis and the RANKL/OPG ratio [11]. We also discovered that FDC-SP secreted by follicular dendritic cells may act on germinal center B cells and participate in the modulation of IgA generation in the tonsils in immunoglobulin A nephropathy (IgAN) [12]. In the tonsils of IgAN patients, FDC-SP expression significantly decreased and was negatively correlated with increased IgA production [13]. It serves as a desirable candidate immunomodulator and offers IgAN treatment options. FDC-secreted protein (FDC-SP) was proved to be specific markers of FDC and related tumor [14]. Meanwhile, TNF- and IL-1 have been shown to increase transcription of the human FDC-SP gene promoter by targeting YY1, GATA, C/EBP2, and C/EBP3 [15, 16]. Several malignancies have been known to arise and progress as a result of aberrant FDC-SP expression. It may encourage ovarian cancer cells to invade and spread [17]. On the one hand, metastasis is the primary cause of morbidity and mortality for renal cancer patients. Previous studies have reported that the overexpression of FDC-SP is related to tumorigenesis and cancer metastasis. Immunotherapy, on the other hand, is an important adjunct to current advanced or metastatic renal cancer. As immunological responses are linked to FDCSP, we hypothesize that it could be used as an immune-related prognostic marker for renal cancer. Up to the present, the role of FDC-SP was poorly understood in RCC. Therefore, it is necessary to study the role of FDCSP in the occurrence and development of renal cancer. However, there have been no studies of this gene in renal cancer.

With the help of bioinformatics, we set out to examine and comprehend the connections between the expression of the FDC-SP and its clinicopathological and prognostic relevance, as well as the underlying molecular pathways and immune cell infiltration in RCC. The significance of FDC-SP in the diagnosis and prognosis of RCC was another goal of our research, since it might offer a novel target for the therapy of renal cancer.

## Methods

### FDC-SP expression level comparison

From the Cancer Genome Atlas (TCGA) database, we obtained the mRNA expression profiles. With the help of these data, it was possible to examine the expression of FDC-SP in 33 different types of human cancer, 539 RCC tissues, 72 normal renal tissues, and 72 RCC tissues along with their paired adjacent problems.

### Atlas of human protein (HPA)

The HPA used transcriptome and proteomics to produce a lot of protein atlases, including the tissue atlas and pathology atlas. We took pictures of RCC and healthy tissue stained with HPA immunohistochemistry.

### Immunohistochemistry

All tissues were fixed in 4% paraformaldehyde, paraffin-embedded, and cut into 4 μm sections. The sections were deparaffinized and rehydrated, antigen retrieval was performed, and 3% H2O2 was used to neutralize endogenous peroxidase. Samples were blocked for 30 min at room temperature in phosphate buffered saline (PBS) (pH 7.4) containing 5% bovine serum albumin. After that, the sections were treated with goat anti-rabbit immunoglobulin G antibody at a 1:100 dilution at 4 °C overnight with FDC-SP antibody (Zhongshan Golden Bridge, Beijing, China). A DAB substrate was used to detect immunoreactivity (Zhongshan Golden Bridge). The tissues were then mounted, counterstained with hematoxylin, and examined under a microscope.

### Cancer stage and FDCSP correlation analysis

From the Cancer Genome Atlas (TCGA) database, we gathered the clinical information of individuals with RCC. Correlation analysis of FDC-SP and cancer stage, including T stage, pathological stage, histological grade, N stage, M stage, age, gender, and OS event, was performed with R using the ggplot2 package.

### Analysis of differentially expressed genes

Patients with RCC in the TCGA were split into groups with high and low FDC-SP expression based on the median FDC-SP expression score. The R package DESeq2 was used to do the differentially expressed gene (DEG) analysis between these two groups, and adjusted *p* value < 0.05, and |log2-fold-change (FC)|> 2 was set as the thresholds of DEGs. The association between the expression of the top 10 DEGs and FDC-SP was examined using Spearman’s correlation analysis.

### Analysis of functional enrichment

We carried out the following analysis to comprehend the biological pathways and processes that FDC-SP might be involved in. The DEGs were subjected to Gene Ontology (GO) and Kyoto Encyclopedia of Genes and Genomes (KEGG) analyses. Gene Ontology (GO) and Kyoto Encyclopedia of Genes and Genomes (KEGG) [18] analyses were all performed by the ClusterProfiler package in R (4.2.1). The R program clusterProfiler was used to perform a gene set enrichment analysis (GSEA). The c5.all.v7.2. symbols.gmt curated reference genesets from the MgDB file were chosen for GSEA. The enriched pathways were sorted using the normalized enrichment score (NES) and the corrected *p*-value. Furthermore, function or pathway terms with an adjusted *p*-value < 0.05 and false discovery rate (FDR) < 0.25 were considered statistically significantly enriched.

### Analysis of the protein–protein interaction network

Information about the protein–protein interaction network for FDC-SP was retrieved from the STRING database. And the Cytoscape software was used to visualize the PPI network.

### Analysis of immune infiltration

ssGSEA was realized by the GSVA package in R to investigate the correlation between FDC-SP expression, immune cell infiltration, and immune cell biomarkers in RCC. The Wilcoxon rank-sum test was used to compare the levels of immunological infiltration between the groups with high and low FDC-SP expression, and Spearman's correlation analysis was used to examine the relationship between the expression of FDC-SP and these immune cells. Results were deemed statistically significant if the *p*-value was less than 0.05.

### Analysis of the relationships between immune checkpoints and FDC-SP

The Spearman's correlation analysis was used to assess the relationship between immune checkpoints in RCC and the expression of FDC-SP. Results were deemed statistically significant if the *p*-value was less than 0.05.

### Survival analysis

Kaplan–Meier plots were created, and the log-rank test was performed using a survival package. Analyses of Cox regression using univariate and multivariate variables were done to determine how clinical factors affected patient outcomes. In the multivariate Cox regression analysis, prognostic factors with *p* < 0.1 in the univariate Cox regression analysis were included. The forest map was shown using the ggplot2 R package.

### Construction and validation of the nomogram

To predict the overall survival probability, a nomogram was established based on some prognostic factors in Cox analysis. Calibration plots were then utilized to assess the performance of the nomogram. Using R packages, such as the pROC, timeROC, and survival packages, the ROC curve of diagnosis, the time-dependent curve of diagnosis, and nomogram model analysis were produced.

### Statistical analysis

Using R, all statistical evaluations were done. Differences between groups were compared using the Wilcoxon rank-sum test or the Student’s t-test, as appropriate. Correlations were determined using Spearman correlation tests, as appropriate. Kaplan–Meier plots were created, and log-rank tests were performed to identify the significance of the difference between the survival curves. Statistical significance was determined at a two-sided *P* value of 0.05.

## Results

### FDC-SP is expressed more frequently in RCC

**Fig. 1:**
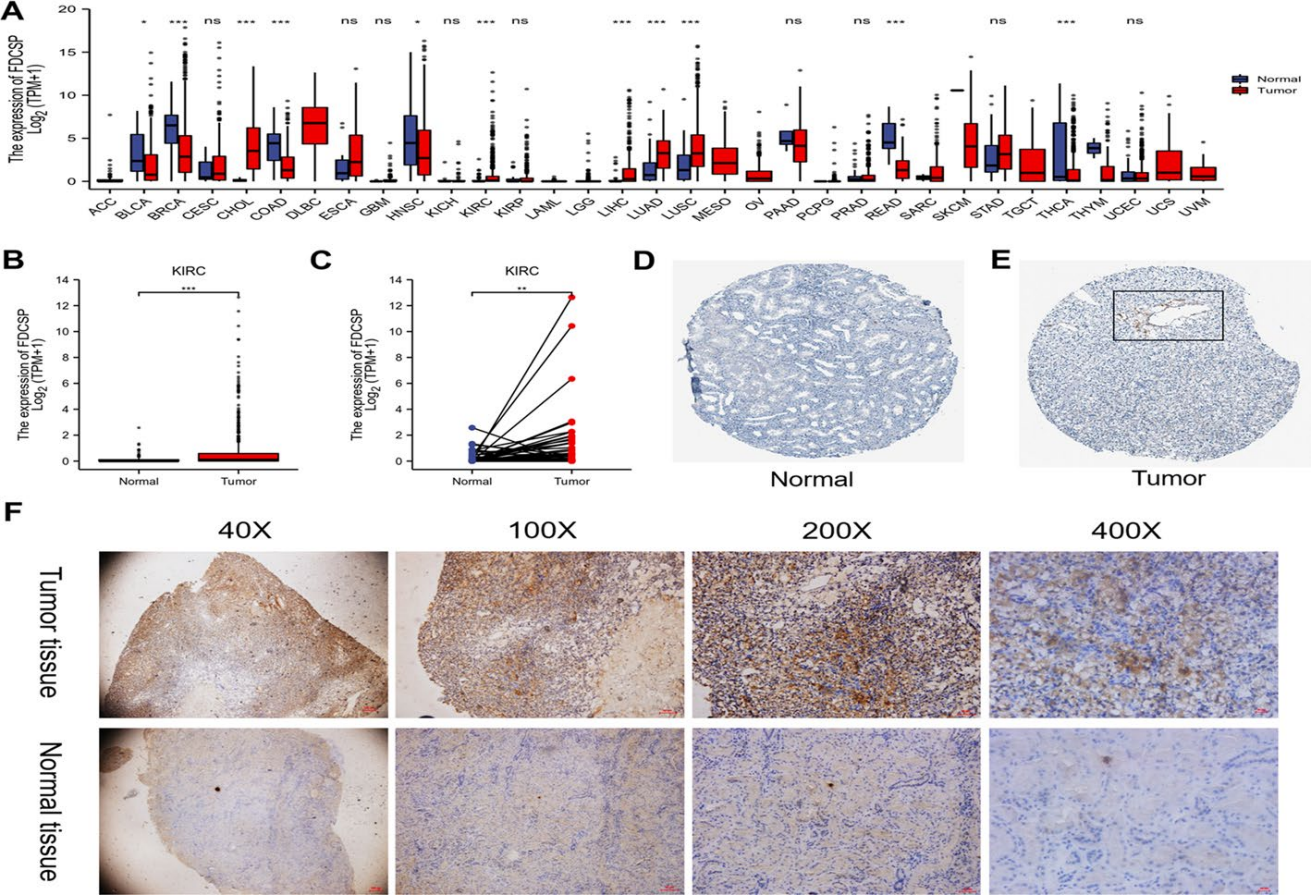
FDC-SP expression levels in several tumor types and RCC. **A** In the TCGA and GTEx databases, 33 different types of cancers were compared to normal tissues for FDC-SP expression. **B** FDC-SP expression in 530 RCC tissues and 72 normal renal tissues according to the TCGA dataset. **C** FDC-SP expression based on TCGA dataset in 72 RCC samples and their associated normal renal tissues. **D**, **E** Using the Human Protein Atlas database, the expression level of FDC-SP was verified (immunohistochemistry). **F** Illustrations showing the expression of FDC-SP in both normal and cancerous tissues of the kidney. 40x, 100x, 200x, and 400× were the initial magnifications. *p < 0.05, **p < 0.01, and ***p < 0.001

To explore the possible roles of FDC-SP, we first analyzed its expression in 33 types of human cancer. FDC-SP was highly expressed in various cancers, including lung adenocarcinoma, lung squamous cell carcinoma, and liver hepatocellular carcinoma (Fig. 1A). RCC samples had considerably greater levels of FDC-SP expression than did healthy renal tissues (*p* < 0.001) (Fig. 1B). In addition, 72 matched renal carcinoma tissues showed significant FDC-SP expression (*p* < 0.01) (Fig. 1C). The HPA database was used to examine the FDC-SP protein levels in RCC. We found that renal cancer tissues had higher amounts of the protein FDC-SP than normal tissues did (Fig. 1D, E). To further identify the expression of FDC-SP, 15 patients with RCC from the Tianjin Third Central Hospital were randomly selected for immunohistochemical staining. The result also confirmed that the level of FDC-SP in renal cancer tissues was higher than that in normal tissues. Representative images are presented in Fig. 1F.

### Clinicopathologic variables and the expression of FDC-SP are associated

**Fig. 2:**
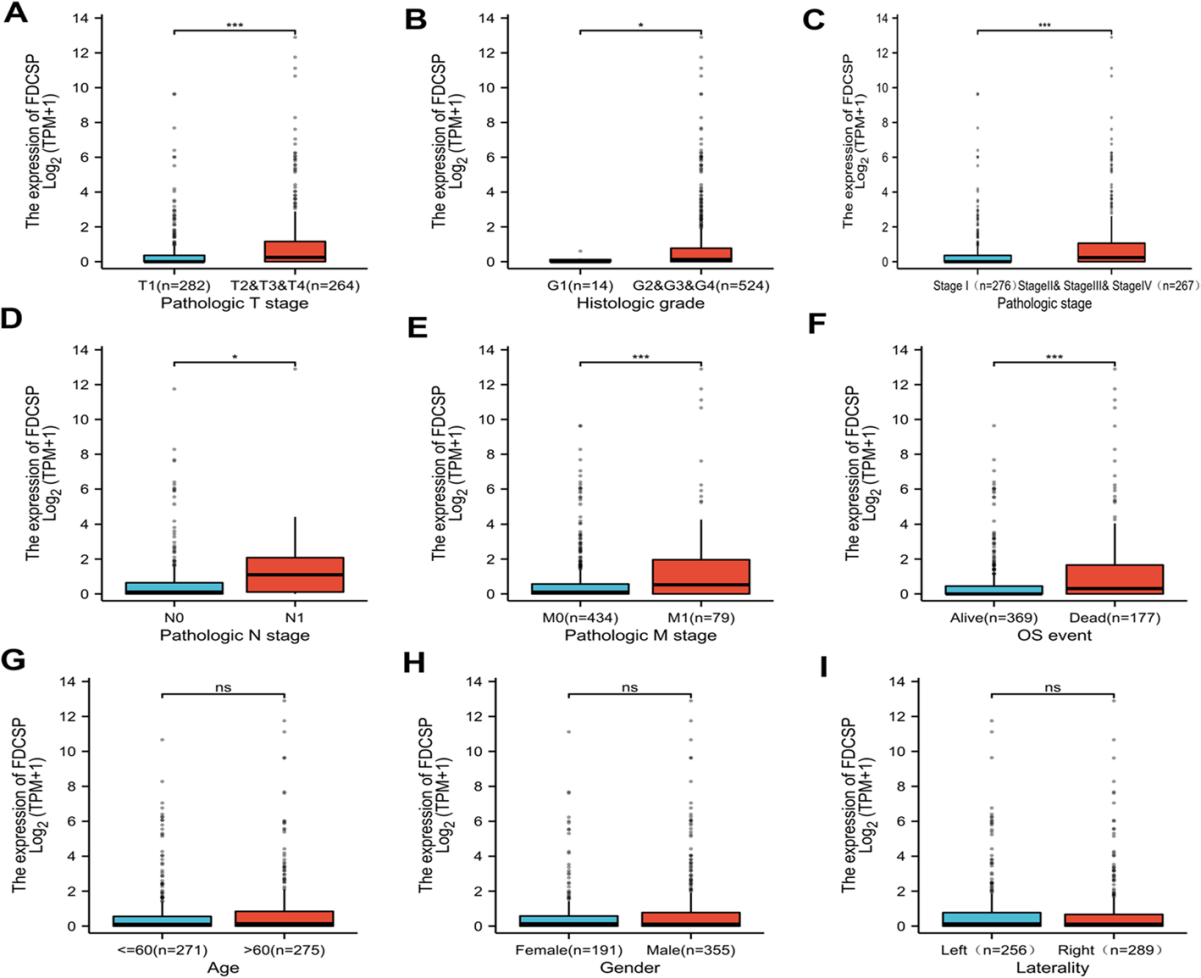
Associations between FDC-SP expression and clinicopathological characteristics. **A** Association between the FDC-SP expression and the T stage of RCC, **B** histologic grade, **C** pathological stage, **D** N stage, and **E** M stage, **F** OS event, **G** Age, **H** Gender, **I** Laterality. *p < 0.05, **p < 0.01, and ***p < 0.001

Table 1 and Fig. 2 demonstrate that the expression of FDC-SP is significantly linked with T stage (*p* < 0.001), histological grade (*p* < 0.05), pathological stage (*p* < 0.001), N stage (*p* < 0.05), M stage (*p* < 0.001), OS event (*p* < 0.001), and is independent of gender, age, and laterality. As for the clinicopathological differences between the groups with high and low expression of FDC-SP, the results of the univariate logistic regression analyses revealed that there were differences in T stage (odds ratio [OR] 1.877, 95% CI 1.312–2.699, *p* < 0.001), N stage ([OR] 3.289, 95% CI 1.110–12.029, *p* = 0.044), M stage ([OR] 2.733, 95% CI 1.257–2.556, *p* = 0.001), pathological stage ([OR] 1.790, 95% CI 1.639–4.687, *p* < 0.001), and histological grade ([OR] 1.427, 95% CI 1.011–2.018, *p* = 0.044) (Table 2). The T stage (*p* < 0.001), N stage (*p* < 0.001), M stage (*p* < 0.001), age (*p* < 0.001), pathologic stage (*p* < 0.001), and FDCSP (*p* < 0.001) were associated with the poor prognosis of RCC in the Cox regression model, according to univariate Cox regression (Table 3). The multivariate Cox regression includes every variable from the univariate Cox regression. T stage (*p* = 0.037), age (*p* = 0.029), and pathologic stage (*p* = 0.012) were identified as independent predictive variables for OS.

**Table 1:** The Cancer Genome Atlas lists the clinicopathological traits of groups with high and low FDC-SP expression (TCGA)

Characteristics	Low expression of FDCSP	High expression of FDCSP	*p* value
n	266	266	
Pathologic T stage, n (%)			0.006
T1	153 (28.8%)	119 (22.4%)	
T2	36 (6.8%)	33 (6.2%)	
T3	74 (13.9%)	106 (19.9%)	
T4	3 (0.6%)	8 (1.5%)	
Histologic grade, n (%)			< 0.001
G1	10 (1.9%)	4 (0.8%)	
G2	122 (23.3%)	106 (20.2%)	
G3	109 (20.8%)	97 (18.5%)	
G4	21 (4%)	55 (10.5%)	
Pathologic stage, n (%)			0.002
Stage I	150 (28.4%)	116 (21.9%)	
Stage II	30 (5.7%)	27 (5.1%)	
Stage III	59 (11.2%)	64 (12.1%)	
Stage IV	27 (5.1%)	56 (10.6%)	
Pathologic N stage, n (%)			0.036
N0	125 (48.8%)	115 (44.9%)	
N1	4 (1.6%)	12 (4.7%)	
Pathologic M stage, n (%)			< 0.001
M0	224 (44.8%)	197 (39.4%)	
M1	24 (4.8%)	55 (11%)	
OS event, n (%)			< 0.001
Alive	201 (37.8%)	156 (29.3%)	
Dead	65 (12.2%)	110 (20.7%)	
Age, n (%)			0.386
< = 60	137 (25.8%)	127 (23.9%)	
> 60	129 (24.2%)	139 (26.1%)	
Gender, n (%)			0.318
Female	88 (16.5%)	99 (18.6%)	
Male	178 (33.5%)	167 (31.4%)	
Laterality, n (%)			0.830
Left	124 (23.4%)	126 (23.7%)	
Right	142 (26.7%)	139 (26.2%)

**Table 2:** Logistic regression analysis of the correlation between FDC-SP expression and clinicopathological features

Characteristics	Total (N)	Odds ratio (OR)	*p* value
T stage (T3&T4 vs. T1&T2)	530	1.877 (1.312–2.699)	< 0.001
N stage (N1 vs. N0)	255	3.289 (1.110–12.029)	0.044
M stage (M1 vs. M0)	498	2.733 (1.639–4.687)	< 0.001
Age (> 60 vs. < = 60)	530	1.163 (0.827–1.636)	0.385
Pathologic stage (Stage III&Stage IV vs. Stage I&Stage II)	527	1.790 (1.257–2.556)	0.001
Gender (Male vs. Female)	530	0.847 (0.592–1.211)	0.363
Race (Asian&Black or African American vs. White)	523	0.863 (0.509–1.458)	0.583
Histologic grade (G3&G4 vs. G1&G2)	522	1.427 (1.011–2.018)	0.044
Laterality (Right vs. Left)	529	0.978 (0.695–1.376)	0.898

**Table 3:** Clinical pathological parameters for RCC patients after univariate and multivariate analyses

Characteristics	Total (N)	Univariate analysis		Multivariate analysis	
		Hazard ratio (95% CI)	*p* value	Hazard ratio (95% CI)	*p* value
T stage	539				
T1	278	Reference			
T2	71	1.515 (0.908–2.526)	0.112	0.177 (0.035–0.903)	**0.037**
T3	179	3.354 (2.373–4.742)	** < 0.001**	0.432 (0.117–1.601)	0.209
T4	11	10.829 (5.467–21.451)	** < 0.001**	0.446 (0.094–2.122)	0.311
N stage	257				
N0	241	Reference			
N1	16	3.453 (1.832–6.508)	** < 0.001**	1.433 (0.492–4.169)	0.509
M stage	506				
M0	428	Reference			
M1	78	4.389 (3.212–5.999)	** < 0.001**	0.429 (0.040–4.615)	0.485
Age	539				
< = 60	269	Reference			
> 60	270	1.765 (1.298–2.398)	** < 0.001**	1.661 (1.052–2.623)	**0.029**
Histologic grade	531				
G1	14	Reference			
G2	235	7,510,356.751 (0.000-Inf)	0.994	3,980,595.814 (0.000-Inf)	0.996
G3	207	14,161,426.542 (0.000-Inf)	0.993	6,771,509.761 (0.000-Inf)	0.996
G4	75	38,204,822.146 (0.000-Inf)	0.993	7,718,630.236 (0.000-Inf)	0.996
Pathologic stage	536				
Stage I	272	Reference			
Stage II	59	1.207 (0.650–2.241)	0.551	4.543 (0.707–29.173)	0.111
Stage III	123	2.705 (1.800–4.064)	** < 0.001**	3.972 (0.986–16.005)	0.052
Stage IV	82	6.692 (4.566–9.808)	** < 0.001**	27.692 (2.086–367.529)	**0.012**
Gender	539				
Female	186	Reference			
Male	353	0.930 (0.682–1.268)	0.648		
FDCSP	539				
Low	269	Reference			
High	270	1.809 (1.325–2.468)	** < 0.001**	0.991 (0.621–1.582)	0.970

The significance of bold is *P* < 0.05

### Analysis of RCC and PPI networks to identify DEGs

**Fig. 3:**
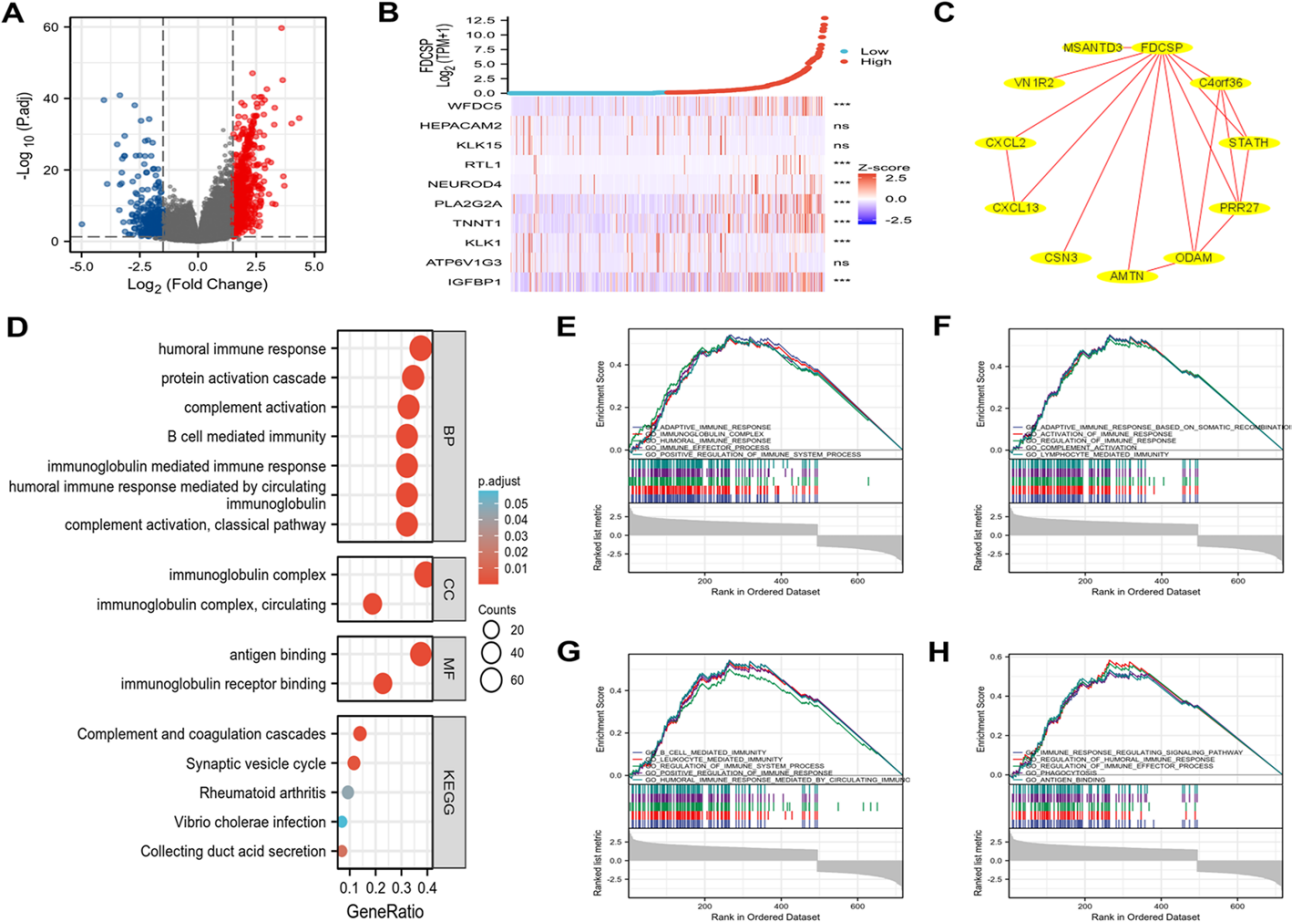
The outcomes of the enrichment analysis and differentially expressed gene (DEG) analysis of FDCSP in RCC. **A** The DEGs volcano plot. The DEGs that have been markedly up- or down-regulated are represented by blue and red dots, respectively. **B** Heatmap showing the relationship between the top 10 DEGs and FDC-SP expression. **C** A network made up of 10 possible co-interaction proteins and the FDC-SP. **D** DEG analysis using GO and KEGG (www.kegg.jp/kegg/kegg1.html). **E**–**H** The outcomes of the GSEA's enrichment analysis. *p < 0.05, **p < 0.01, and ***p < 0.001

Between the groups with high and low expression levels of FDC-SP, 329 genes were differentially expressed, including 205 upregulated DEGs (39.4%) and 124 downregulated DEGs (60.6%) (adjusted *p* value < 0.05, |Log2-FC|> 2) (Fig. 3A and Additional file 1: Table S1). Following that, the relationship between the top 10 DEGs (including WFDC5, HEPACAM2, KLK15, RTL1, NEUROD4, PLA2G2A, TNNT1, KLK1, ATP6V1G3, and IGFBP1) and FDC-SP is presented in Fig. 3B. We created a PPI network using the online STRING tool and then identified the hub genes to investigate the potential interactions between all identified DEGs. As shown in Fig. 3C, the network of the DEGs was complex, and the top 10 hub genes were CSN3, AMTN, ODAM, PRR27, STATH, C4orf36, MSANTD3, VN1R2, CXCL2, and CXCL13 (Additional file 2: Table S2).

### Analysis of functional enrichment using GO, KEGG, and GSEA

The three main functions considered in the GO enrichment analysis were biological processes, cellular components, and molecular functions. The biological process was primarily composed of B cell-mediated immunity, complement activation, protein activation cascades, and humoral immune responses. Cellular components mainly included immunoglobulin complexes and circulating immunoglobulin complexes. Antigen binding and immunoglobulin receptor binding were the two basic molecular activities. KEGG pathway analysis revealed that rheumatoid arthritis, complement and coagulation cascades, synaptic vesicle cycle, collecting duct acid secretion, and vibrio cholerae infection were among the significantly DEGs-enriched pathways (Fig. 3D and Additional file 3: Table S3). GSEA was then used to compare the groups with high and low FDC-SP expression, and it was discovered that the high FDC-SP expression group had more immune-related biological processes that were significantly enriched. This finding suggests that high FDC-SP expression elevates the immunophenotypic profile in RCC (Fig. 3E–H and Additional file 4: Table S4).

### Immune infiltration and FDC-SP expression are associated

**Fig. 4:**
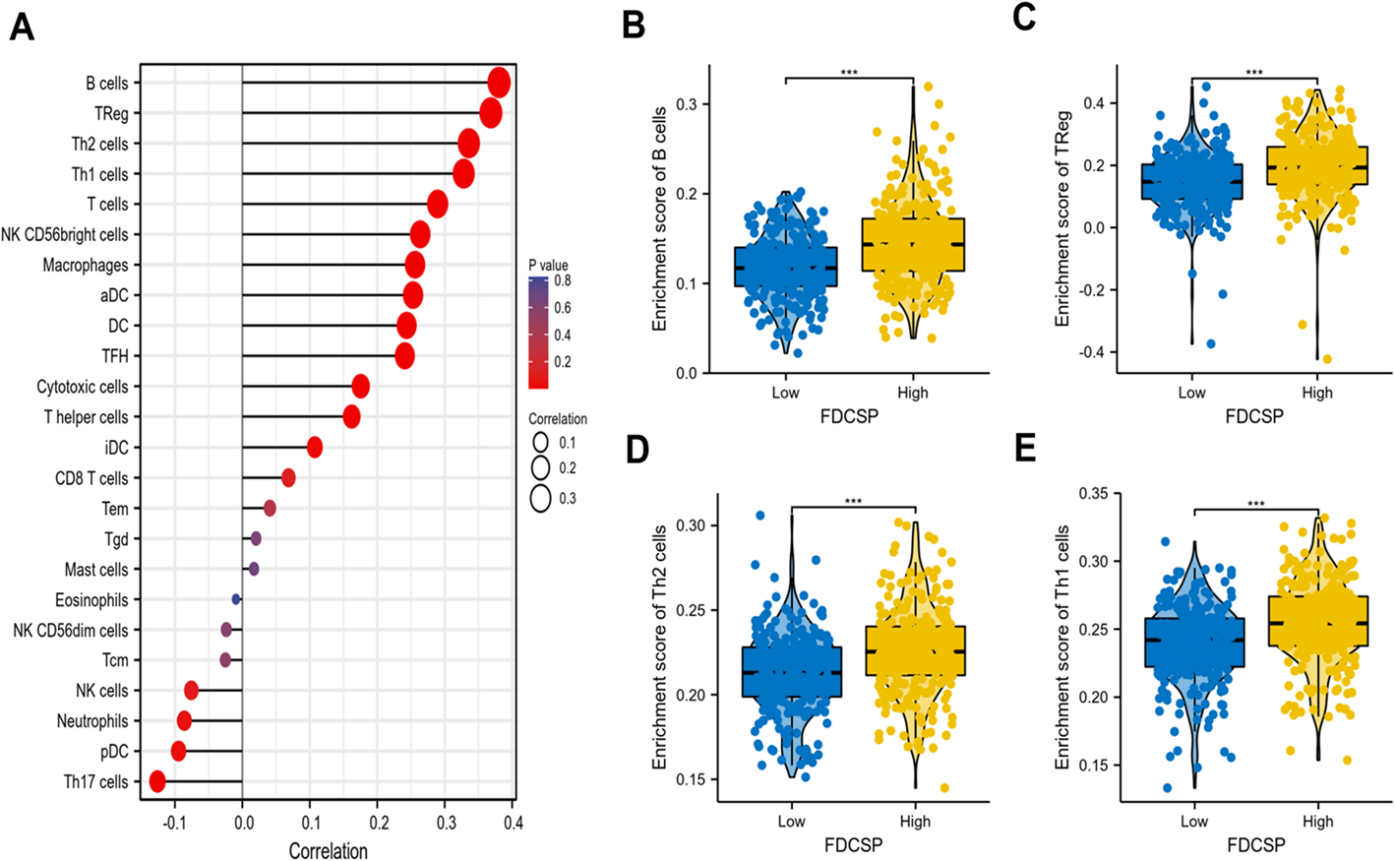
Correlation of FDCSP expression with immune infiltration level. **A** Correlation between the relative abundances of 24 immune cells and the amount of FDC-SP expression. **B**–**E** Comparison of immune infiltration levels between groups with high and low FDC-SP expression, including B cells, Treg, Th2 cells, and Th1 cells. *p < 0.05, **p < 0.01, and ***p < 0.001

**Fig. 5:**
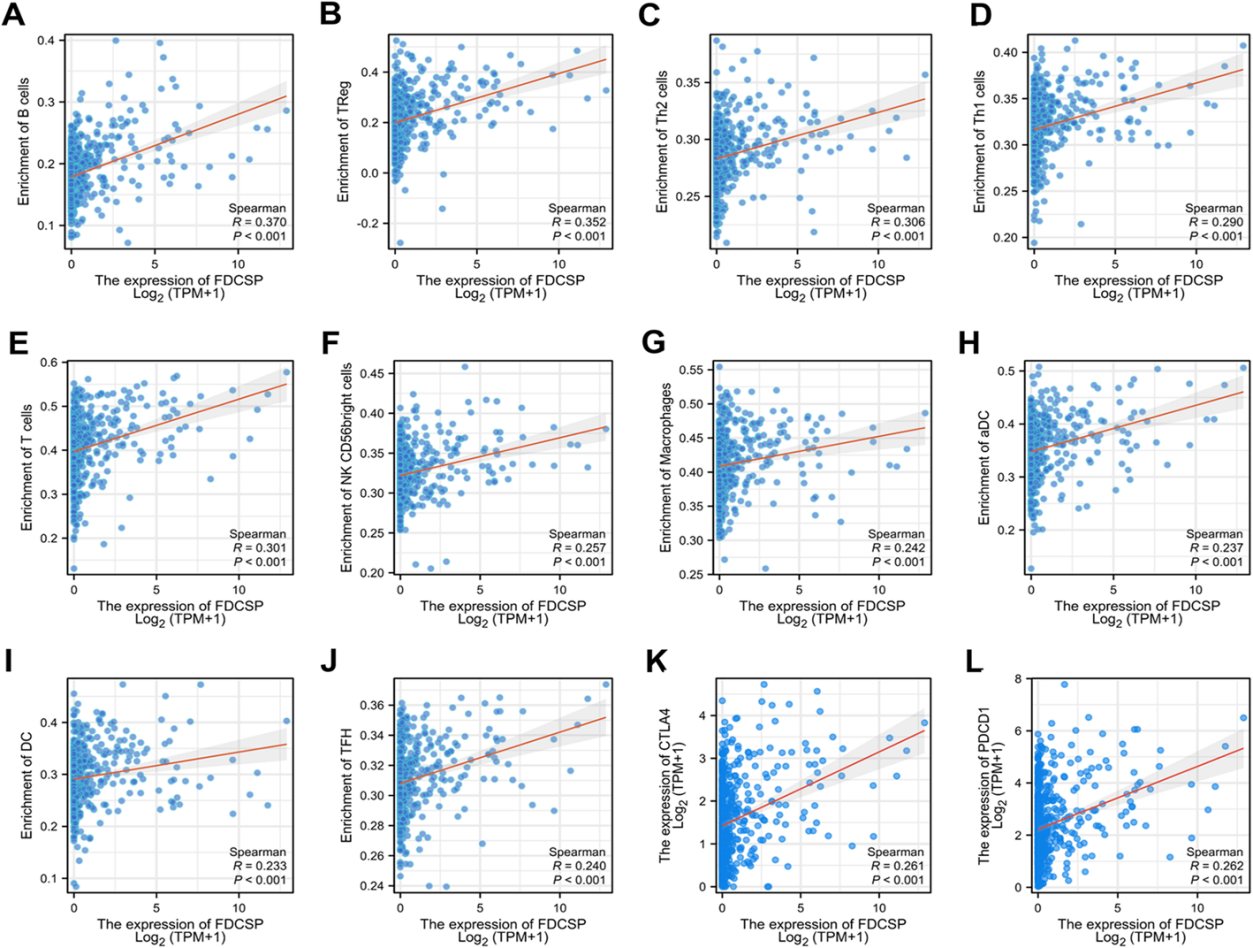
The correlation analysis of FDC-SP with Immune Cells and Immune Checkpoints. **A**–**G** Correlations between the expression of FDC-SP and the relative enrichment scores of immune cells, including B cells, Treg, Th2 cells, Th1 cells, T cells, NK CD56bright cells, Macrophages, aDC, DC, and TFH. The correlation analysis results between the expression levels of FDC-SP and the expression levels of **J** CTLA-4, **K** PDCD-1. *p < 0.05, **p < 0.01, and ***p < 0.001

Natural killer B cells, Treg, Th2 cells, and Th1 cells' levels of immune cell infiltration were all significantly positively linked with the expression of FDC-SP (Fig. 4A). In addition, the enrichment scores of B cells, Treg, Th2 cells, and Th1 cells in the FDC-SP high expression group were markedly higher than those in the FDC-SP low expression group (all *p* < 0.001) (Fig. 4B–E). To further explore the role of FDC-SP in the tumor immune micro-environment, we identified the correlation between FDC-SP expression and immune cells in RCC. FDC-SP significantly positively correlated with B cells, Treg, Th2 cells, Th1 cells, T cells, NK CD56bright cells, Macrophages, aDC, DC, and TFH (Fig. 5A–J).

### Relationship between FDC-SP expression and immune checkpoints in RCC

As is well known, tumor immune escape is linked to crucial immunological checkpoint proteins, including CTLA-4 and PDCD-1. Evaluation of the interrelationship between FDC-SP, CTLA-4, and PDCD-1 was done using the TCGA databases. We found a positive correlation between CTLA-4, PDCD-1, and FDC-SP expression levels (Fig. 5K–L).

### Prognostic value of FDC-SP in RCC

**Fig. 6:**
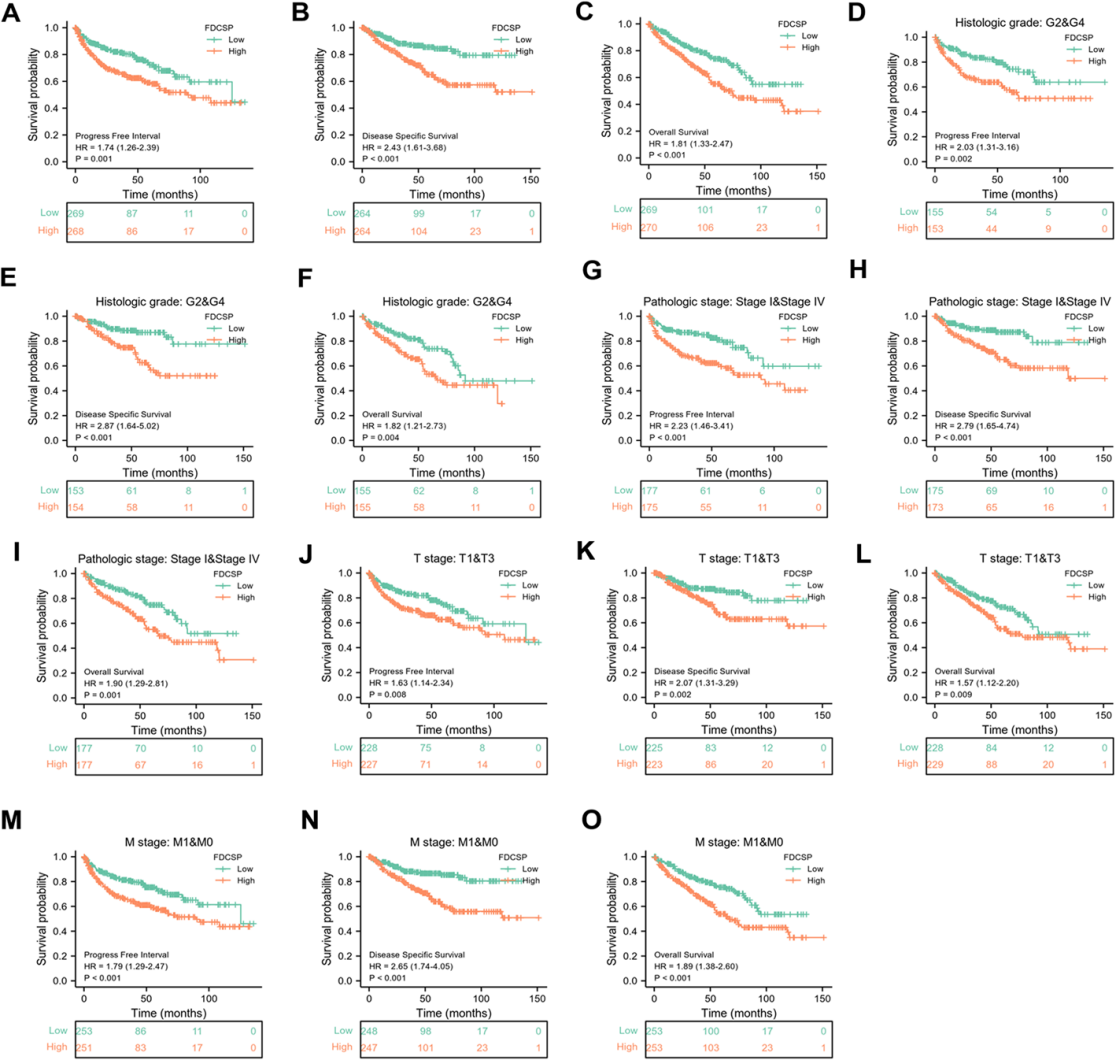
Prognostic values of FDC-SP expression in patients with RCC. **A–C** The prognostic value of FDC-SP in PFI, DSS, and OS of RCC. **D**–**O** DFI, DSS, and OS survival curves of G2 and G4, stage I and IV, T1 and T4, M0 and M1 between high- and low-FDC-SP patients with RCC

Using the Kaplan–Meier approach, it was estimated that there was a link between FDC-SP expression and the prognosis of RCC patients. According to K-M survival curve analysis, RCC patients with high FDC-SP expression had substantially lower PFI, DSS, and OS values compared to those with low FDC-SP expression levels (PFI: HR = 1.74, 95% CI 1.26–2.39, *p* = 0.001; DSS: HR = 2.43, 95% CI 1.61–3.68, *p* < 0.001; OS: HR = 1.81, 95% CI 1.33–2.47, *p* < 0.001) (Fig. 6A–C). A worse outcome was associated with overexpression of FDC-SP. Then, we assessed the associations between prognosis and FDC-SP expression in various subgroups. Patients with high FDC-SP expression had noticeably worse prognoses in several subgroups, including G2 and G4, stage I and IV, T1 and T3, and M0 and M1 (all *p* < 0.05), regardless of OS, DSS, or PFI (Fig. 6D–O).

### The importance of FDC-SP expression in diagnosis and prognostication

**Fig. 7:**
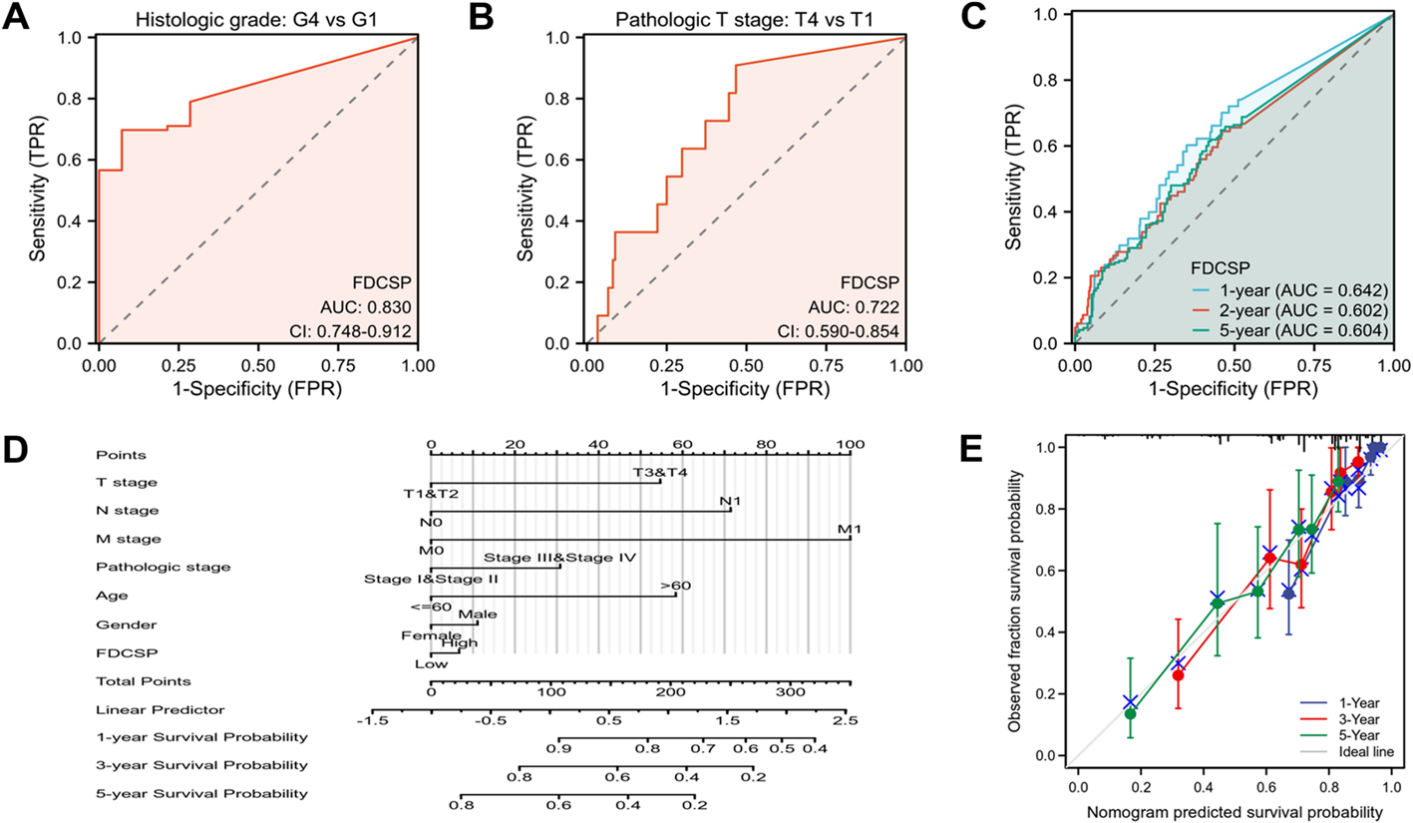
Value of FDC-SP expression in diagnostic and prognosis prediction. **A**, **B** The FDC-SP diagnostic ROC curve. **C** Time-dependent ROC curve of FDC-SP. **D** A nomogram for estimating the overall survival rates of RCC patients after one, three, and five years. **E** The nomogram's calibration curves for predicting the one-, three-, and five-year overall survival rates of RCC patients

FDC-SP expression demonstrated the ability to precisely distinguish high-grade or high-stage renal cancer by the ROC curve of diagnosis (AUC = 0.830, 0.722) (Fig. 7A, B). To forecast survival rates at one, two, and five years, a time-dependent survival ROC curve of FDC-SP was set up. These AUC values were all greater than 0.6, considered suitable for prediction (Fig. 7C). Age, pathologic phases, gender, T stages, M stages, N stages, and FDC-SP expression levels were all incorporated as parameters in the nomogram model that was created (Fig. 7D). An increased total number of points on the nomogram was linked to a worse prognosis. In clinical practice, the nomogram model can be used to forecast a patient's chances of survival at one, three, and five years. The nomogram's bootstrap-corrected C-index was 0.769 (95% CI 0.745–0.792), demonstrating a reasonable level of predictive accuracy for OS in RCC patients. Additionally, calibration curves were employed to calculate the nomogram's predictive power (Fig. 7E).

## Discussion

FDC-SP was initially isolated from human reactive tonsils in 2002. Previous studies on this gene were focused on its mediator function of B lymphoma cells. It can specifically connect to active B cells and act as a regulator of antibody responses. However, it was unclear how this gene worked. According to the study, the majority of the more than 30 sequences that match its sequence come from libraries of fetal tissue or tumor cells. This suggests that it might be crucial for cancer or embryonic development. The study found that the FDC-SP gene is overexpressed in several tumor types, including breast and ovarian cancers as well as endometrial cancer. On the other hand, it was hardly ever expressed in the equivalent benign lesions or normal tissues. FDC-SP promotes EOC cell migration and invasiveness and decreases cell–cell adhesion. To date, the role of the FDC-SP in the RCC has not been investigated. Thus, we decided to employ bioinformatics techniques to comprehend the biological function and potential regulatory mechanisms of FDC-SP in renal clear cell carcinoma in the light of the paucity of research on FDC-SP in malignancies.

In this study, we used the TCGA database to examine the expression of FDC-SP in renal malignancies and discovered that, in contrast to normal tissues, FDC-SP was substantially expressed in renal cancer. Furthermore, our findings demonstrated a negative correlation between high FDC-SP expression and unfavorable clinicopathologic variables such as T stage, pathological stage, and histological grade. Additionally, we discovered that higher FDC-SP expression is a standalone predictive biomarker of OS in patients with renal clear cell carcinoma and that FDC-SP has a clear reference value for the diagnosis and prognosis of renal cancer. We also evaluated the link between FDC-SP and therapeutic targets and immunological checkpoints for renal cancer. Currently, targeted therapy and immunotherapy are of particular interest for patients with advanced or metastatic renal cancer. The findings demonstrated that FDC-SP was positively correlated with PDCD-1 and CTLA-4, suggesting that targeting FDC-SP may increase the effectiveness of immune checkpoint inhibitors. These findings strongly imply that FDC-SP can be used as a biomarker for early diagnosis and prognosis monitoring in renal cell carcinoma patients, and as a novel molecular target for the treatment of renal cancer.

Tumor cells exhibit unchecked growth and multiplication and a special immunological escape mechanism that prevents them from being eliminated by immune cells and natural killer cells [19]. We used GO functional enrichment and KEGG pathway analysis in this study to investigate the molecular mechanisms underlying the progression of RCC and poor prognosis caused by FDC-SP, and we discovered that the immune response and complement activation were most significantly enriched in the FDC-SP high-expression group. GSEA also supported this judgment. It has been previously shown that increased FDC-SP expression is linked to increased cell viability and aggressiveness in breast cancer cells, which can phosphorylate Akt at serine 473 and reduce E-cadherin expression. To investigate the precise function and signaling pathways of FDC-SP in renal cancer, these findings require additional experimental confirmation.

The tumor microenvironment (TME) is a factor in the development of cancer [20, 21]. The extracellular matrix, inflammatory mediators, immune cells, and cancer cells all contribute to the development, metastasis, and clinical prognosis of tumors [22]. The effect of tumor infiltrating immune cells on tumor development and prognosis has been demonstrated in solid malignancies and is influenced by the type, density, and location of immune cells [23, 24]. Screening immune cells infiltrating renal cancer can help predict how the disease will respond to immune checkpoint inhibitors. The study found that FDC-SP overexpression was positively correlated with the infiltration of B cells, T cells, CD8 T cells, and TEM cells and negatively correlated with the infiltration of NK cells and pDCs. Given that high FDC-SP expression is linked to the immune response and B cell activation, we next assessed the correlation between FDC-SP expression and the levels of immune infiltration cells. Renal cancer immunotherapy has been shown to be effective, and NK cells and CD8 T cells have both been shown to combat the disease. These findings imply that kidney cancer and tumor immune microenvironment progression may be significantly influenced by FDC-SP overexpression.

Our study's findings, which included a considerable upregulation of FDC-SP expression, were linked to renal cancer's poor prognosis and clinicopathological characteristics. The reference value of FDC-SP for the diagnosis and prognosis of renal carcinoma is obvious. Targeting FDC-SP may increase the efficacy of targeted therapy and immunotherapy, offering new ideas for the diagnosis and treatment of renal cancer. It may alter tumor growth by modulating immune infiltration and the immune response to renal cancer.

Our research has currently shed some light on the connection between FDC-SP and the clinical, pathological, and prognostic features of renal cancer, but there are always some restrictions. First off, the study's data are small and singular, coming from online databases. Additionally, some crucial clinical information is missing, necessitating multiple data cross-validations and the gathering of substantial clinical data. Second, to confirm the role of FDC-SP in renal cancer and the underlying molecular mechanisms, a variety of tests are required in vitro. These issues merit more research and investigation.

In conclusion, FDC-SP could be a potential therapeutic target as well as a diagnostic and prognostic biomarker in RCC. It will help to improve the rates of early diagnosis and the poor prognosis.

## Supplementary Information

**Additional file 1.** 329 DEGs associated with FDC-SP.

**Additional file 2.** Annotation of 10 functional partner genes of FDC-SP in STRING database.

**Additional file 3.** Functional enrichment for FDC-SP-related DEGs along GO and KEGG pathways.

**Additional file 4.** Analysis of GSEA enrichment for DEGs connected to FDCSP.

**Additional file 5.** Other FDC-SP expression level image data.

## Data Availability

The original contributions presented in the study are included in the article/supplementary material, further inquiries can be directed to the corresponding author/s.
