# Supplementary figures and images for: FDC-SP as a diagnostic and prognostic biomarker and modulates immune infiltrates in renal cell carcinoma
Source: BMC Bioinformatics. 2023 Mar 10;24:91. doi: 10.1186/s12859-023-05215-1 (PMC10007807; doi:10.1186/s12859-023-05215-1)

**Other FDC-SP expression level image data**

**
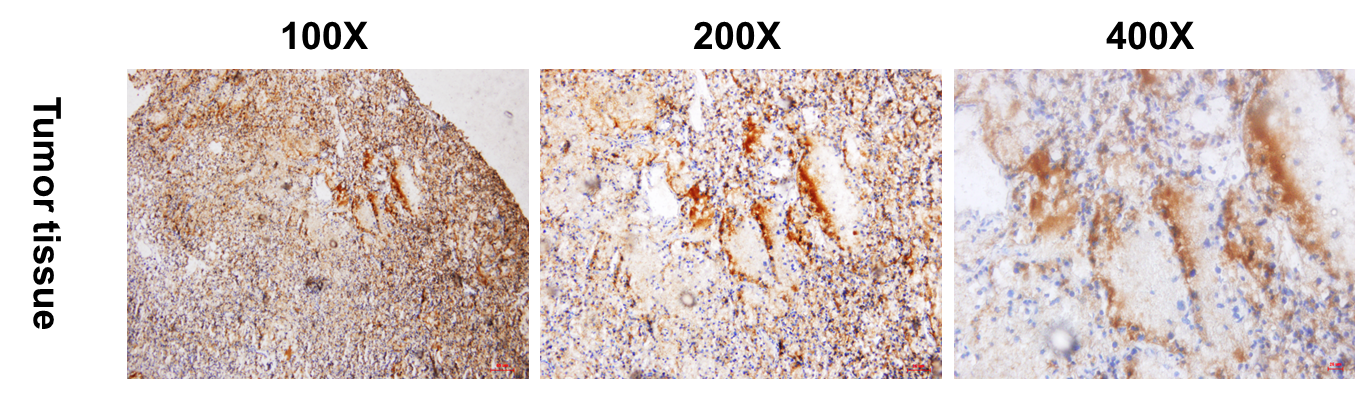
**

Supplement: Supplementary file 5 — Additional file 5. Other FDC-SP expression level image data. [file 12859_2023_5215_MOESM5_ESM.docx]
